# Immunophenotypes and Immune Markers Associated with Acute Promyelocytic Leukemia Prognosis

**DOI:** 10.1155/2014/421906

**Published:** 2014-06-19

**Authors:** Fang Xu, Chang-Xin Yin, Chun-Li Wang, Xue-Jie Jiang, Ling Jiang, Zhi-Xiang Wang, Zheng-Shan Yi, Kai-Kai Huang, Fan-Yi Meng

**Affiliations:** ^1^Hematology Department, Nanfang Hospital, Southern Medical University, Guangzhou 510515, China; ^2^Hematology Department, Mianyang Center Hospital, Mianyang 621000, China

## Abstract

CD2+, CD34+, and CD56+ immunophenotypes are associated with poor prognoses of acute promyelocytic leukemia (APL). The present study aimed to explore the role of APL immunophenotypes and immune markers as prognostic predictors on clinical outcomes. A total of 132 patients with de novo APL were retrospectively analyzed. Immunophenotypes were determined by flow cytometry. Clinical features, complete remission (CR), relapse, and five-year overall survival (OS) rate were assessed and subjected to multivariate analyses. The CD13+CD33+HLA-DR-CD34− immunophenotype was commonly observed in patients with APL. Positive rates for other APL immune markers including cMPO, CD117, CD64, and CD9 were 68.7%, 26%, 78.4%, and 96.6%, respectively. When compared with patients with CD2− APL, patients with CD2+ APL had a significantly higher incidence of early death (50% versus 15.7%; *P* = 0.016), lower CR rate (50% versus 91.1%; *P* = 0.042), and lower five-year OS rate (41.7% versus 74.2%; *P* = 0.018). White blood cell (WBC) count before treatment was found to be the only independent risk factor of early death, CR failure, and five-year mortality rate. Flow cytometric immunophenotype analysis can facilitate prompt APL diagnosis. Multivariate analysis has demonstrated that WBC count before treatment is the only known independent risk factor that predicts prognosis for APL in this study population.

## 1. Introduction

Acute promyelocytic leukemia (APL) is a myeloid leukemia subtype associated with a high mortality rate in newly diagnosed patients. Prompt diagnosis and proper use of all-transretinoic acid (ATRA) are needed to prevent death and improve overall prognosis. Most diagnostic tests for APL, including chromosome examination, polymerase chain reaction, and fluorescent in situ hybridization, are time-consuming. Flow cytometric immunophenotypic analysis has gained attention as an effective and rapid diagnostic tool for APL. It is well documented that CD2+, CD56+, and CD34+ APL immunophenotypes are associated with lower overall survival (OS) rate, shorter remission, decreased incidence of remission, and increased incidence of early death, respectively [1–4]. However, the relationship between these APL immunophenotypes and disease prognosis has not been fully explored. The present study investigated the efficacy of flow cytometric analysis for detecting CD2+, CD56+, and CD34+ APL immunophenotypes and other immune markers as diagnostic tools and predictors of early death and long-term prognosis in APL.

## 2. Materials and Methods

### 2.1. Patients

A total of 132 patients with de novo APL, who were hospitalized at the Nanfang Hospital (Guangzhou, China) between January 2003 and December 2012, were retrospectively enrolled in this study. Written informed consent was obtained from all patients, and the study protocol underwent thorough review and approval process at the hospital's ethics committee. Informed consent was obtained from all patients included in the study.

### 2.2. Flow Cytometric Analysis

Bone marrow samples from all patients were collected in EDTA tubes before treatment. Leukemia cell analysis was performed by standard immunofluorescence methods using monoclonal antibodies directed against cMPO, CD33, CD13, CD117, CD9, CD64, CD11b, HLA-DR, CD2, CD19, CD15, CD71, CD34, and CD56. All samples were studied by direct immunofluorescence. All antibodies were purchased from BD Biosciences (San Jose, USA), and flow cytometric analyses were performed with a FACSCanto II flow cytometer.

### 2.3. Treatment

All patients received induction and maintenance treatment according to guidelines set by the Hematological Society of the Chinese Medical Association [5]. When a diagnosis of APL was suspected, ATRA (30 mg/m^2^/d) was given as induction treatment, as early as possible, until complete remission (CR) was achieved. Thirty-six patients simultaneously received arsenic trioxide (0.15 mg/kg/day for 14 days). For patients with a white blood cell (WBC) count <5 × 10^9^/L, chemotherapy was given until the WBC count increased to above the normal level. For other patients, chemotherapy was usually given as soon as ATRA was initiated. Chemotherapy comprised treatment with idarubicin (8 mg/m^2^/day on days 1, 3, and 5), daunorubicin (45 mg/m^2^/day on days 1, 3, and 5), homoharringtonine (2 mg/m^2^/day on days 1–5), and/or cytarabine (100 mg/m^2^/days on days 1–7). Hydroxycarbamide was given before or after chemotherapy to decrease WBC count. Induction was followed by three consolidation cycles with anthracycline-based chemotherapy. Maintenance treatment continued for two years and comprised at least five cycles of three months each. Each cycle comprised ATRA (30 mg/m^2^/day for 28 days), arsenic trioxide (0.15 mg/kg/day for 15 days), and oral methotrexate (MTX) (6 mg/m^2^ qw for four weeks), combined or not with 6-mercaptopurine (6 MP) (75 mg/m^2^/day for 28 days).

### 2.4. Statistical Analyses

All statistical analyses were performed using SPSS v.17.0 software (SPSS Inc., Chicago, USA). Clinical features are presented as percentages (%) for categorical variables and as mean values ± standard deviation (SD) for normally distributed continuous variables. The *χ* ^2^ test was used to analyze differences in the distribution of categorical variables between patient subsets. The *t*-test or Mann-Whitney test was used to detect differences in the distribution of continuous parametric variables. The Mann-Whitney test was used to analyze differences in the distribution of ranked variables. Multivariate analyses were performed using a binary logistic regression model. *P* values <0.05 were considered statistically significant. A cutoff of >10% was used to quantify the presence of a subpopulation of CD34+ and CD56+ cells, and a cutoff of >20% was used for defining positivity for other antigens. Early death was defined as death during induction therapy or death before achieving complete remission.

## 3. Results

### 3.1. Patient Cohort and Immunophenotypic Analysis

The median age of 132 patients with de novo APL (male: 74, female: 58) enrolled in this study was 31 years (range: 13–67 years). All patients were t (15; 17) or PML-RAR*α* positive. The percentages of patients, who were positive for each tested antigen, are listed in Table 1. Antigens associated with hemopoietic stem cell-like HLA-DR and CD34 were not frequently expressed. HLA-DR was expressed in five of 130 cases (3.8%), while CD34 was expressed in 15 of 129 patients (13.2%). Data for CD9 was collected for 87 patients, of which 96.6% (84 patients) were CD9+. Forty-two of the patients with CD9+ expressed bright CD9, CD2, and CD56. Data for CD2 and CD56 were collected for 101 and 113 patients, respectively, of which 12 (11.9%) and 4 patients (9.3%) were CD2+ and CD56+, respectively (Table 1).

### 3.2. Clinical Features and Prognoses in Patients with CD2+ APL

The present study further evaluated the differences in clinical features and prognoses between patients with CD2+ APL (*n* = 12) and CD2− APL (*n* = 89). Comparisons of baseline clinical features showed no differences in age, gender, and CD56 expression between these two groups. However, WBC counts before treatment in the CD2+ APL group were significantly higher than in the CD2− APL group ([15.06 ± 22.49] × 10^9^/L versus [34.97 ± 57.6] × 10^9^/L; *P* = 0.028). In addition, more patients in the CD2+ APL group expressed CD34 than patients in the CD2− APL group (13.74% versus 3.63%; *P* = 0.006).

The comparison of clinical outcomes showed that patients with CD2+ APL had a significantly higher early death rate (50% versus 15.7%), lower incidence of CR (50% versus 91.1%), and lower five-year OS rate (41.7% versus 74.2%) than patients with CD2− APL. However, five-year relapse rates between these two groups were similar (Table 2).

### 3.3. Multivariate Associations

Multivariate analyses revealed that WBC count before administration of anthracycline-based chemotherapy was an independent risk factor for the occurrence of differentiation syndrome (DS) (*P* = 0.006, OR = 1.022, 95% confidence interval (CI) = 1.006–1.038). WBC count before anthracycline-based chemotherapy also influenced the occurrence of early death (*P* = 0.004, OR = 1.026, 95% CI = 1.008–1.045) and remission failure (*P* = 0.002, OR = 1.028, 95% CI = 1.010–1.046). However, the period from anthracycline-based chemotherapy to ATRA treatment was not an independent risk factor for DS, early death, or remission failure.

### 3.4. Relationship between CD2 Expression and APL Prognosis

Forward stepwise logistic regression analyses were used to measure the influence of CD2, CD34, and CD56 expression and WBC count before treatment on the incidence of DS, early death, remission failure, five-year OS, and five-year relapse. Significant factors (*P* < 0.05) were included in the analyses, while those with *P* values of >0.1 were excluded.

Only CD2, CD34, and CD56 expressions were initially considered for multivariate analysis (Table 3). Analysis revealed that CD2+, CD34+, and CD56+ immunophenotypes were not independent risk factors for DS, remission failure, five-year survival, and five year-relapse. However, study results indicated that CD2 expression might have an impact on early death (*P* = 0.048, OR = 4.333, 95% CI = 1.015–18.508). Subsequently, WBC count before treatment was considered with CD2, CD34, and CD56 expression for multivariate analysis. CD2 expression had no effect on early death, but WBC count before treatment was the only independent risk factor for DS, remission failure, five-year OS, and five-year relapse (Table 4).

## 4. Discussion

The present study involved a flow cytometric immunophenotypic analysis on 132 patients with de novo APL to determine if any immune markers could be used as diagnostic tools or prognostic predictors for APL. The study data are consistent with previous reports, demonstrating that CD13+CD33+HLA-DR-CD34− is a classic immune pattern for APL [1, 6–10]. Other antigens may be important for differential diagnosis. In contrast to myeloid leukemia, this study showed that CD33 expression in all patients with APL was bright, CD13 expression was dim to bright, cMPO expression was dim to moderate, and CD117 expression was generally dim. CD64 is usually expressed by promyelocytes through metamyelocytes; CD64 expression is often bright and specific in patients diagnosed with acute monocytic leukemia [11, 12]. CD64 expression is common in APL but is highly variable. CD9 is also often detected in APL, and 96.6% of the patient cohort in this study was CD9+ with moderate to bright expression. The significance of CD9+ prevalence in APL is still not clear. However, it has been suggested that CD9 may monitor minimal residual disease (MRD) [13, 14]. Although APL diagnoses should be established on the basis of molecular genetic tests, the findings from the current study suggest that, besides the distinct immunophenotypes, other not frequently expressed myeloid antigens, including CD64 and CD9, can facilitate initial and prompt diagnoses of APL.

CD2 is a T cell antigen that is often expressed on APL cells. Previous research has shown that CD2+ immunophenotypes in patients with APL are associated with leukocytosis and the hypogranular M3v phenotype, as well as a higher probability of thrombosis [2, 6, 9, 15]. However, the relationship between CD2 expression and clinical outcomes has not been completely determined. Kaito et al. found that patients with CD2+ APL had lower CR and OS rates than patients with CD2− APL [3]. In the present study, patients with CD2+ APL had higher WBC counts and higher CD34+ rates than patients with CD2− APL. Ninety-one cases were analyzed to investigate the correlation between CD2, early death, and long-term outcomes. Compared with patients with CD2− APL, incidence of early death in patients with CD2+ APL was higher, but there was no significant difference in five-year relapse rates. These data are consistent with those of Kaito et al., suggesting that CD2 expression influences CR and OS rates in patients with APL. However, the CR rate determined for patients with CD2− APL in the current study was higher (91.1% versus 87%), CR rates in patients with CD2+ APL were comparable (approximately 50%), and early death rate was lower (50% versus 66.7%) [3]. The reason for this discrepancy may be attributed to the fact that the patients in our study received arsenic trioxide (ATO) during induction and maintenance treatments. Lou et al. reported that ATO-based combination therapy may eliminate the difference in OS between high risk and intermediate/low risk APL and improve relapse-free survival in de novo APL patients with or without additional chromosome abnormalities (ACAs), while ACAs had no impact on prognosis [16, 17].

The study determined the use of CD2+ immunophenotype as a prognostic predictor for patients with APL. Previous studies have demonstrated the coexpression of CD2, CD34, and CD56 on APL cells [1, 4]. The present study found that CD34 expression is higher in patients with CD2+ APL than in patients with CD2− APL, while no difference was observed with CD56 expression between the two groups. These data suggest that CD2 expression may be associated with CD34. Moreover, both the research findings and available literature have shown an association between leukocytosis and CD2, CD34, and CD56 expression [1, 4]. Therefore, it was hypothesized that CD2, CD34, and CD56 expression and WBC count before treatment might interact and influence the clinical outcomes of patients with APL.

Although the correlation of CD2 positivity with elevated WBC count and poor survival is well established in univariate analyses [1, 4], data describing the impact of CD2 positivity on outcome in multivariate analyses are limited. To investigate this and identify independent risk factors, a multivariate analysis was designed including CD2, CD34, and CD56. This analysis indicated that CD2 was an independent risk factor for early death, and these data were consistent with those determined by single factor analysis. It also suggested that CD2 could replace CD34 and CD56 in predicting early death. However, once WBC count was considered along with CD2, CD34, and CD56, the results indicated that only WBC count before treatment was an independent risk factor for early death, CR failure, and five-year OS. Therefore, assessing WBC count before treatment may be more important than CD2, CD34, and/or CD56 in predicting APL prognosis. The outcome of APL patients appears to be influenced more by WBC than immunophenotype.

The relationship between these immune markers and clinical outcome is currently being scrutinized. Ahmad et al. reported that CD34+ expression was significantly associated with decreased incidence of molecular remission, increased incidence of early death, and higher WBC count [1]. However, Albano et al. compared CR, OS, and DS between patients with CD34+CD2− APL and CD34−CD2− APL; and there were no significant differences [2]. Nonetheless, both CD2+ and CD34+ immunophenotypes are associated with leukocytosis, so differences in clinical outcomes between patients with CD2+ APL and CD2− APL or CD34+ APL and CD34− APL may be due to WBC counts and not because of CD2+ or CD34+ expression. To examine this possibility, multivariate analysis was used to eliminate any such bias.

WBC and platelet counts are continually being challenged by new molecular markers as possible diagnostic and prognostic tools for APL [18–20]. To date, there are no markers that can completely replace WBC counts in predicting APL prognosis. In fact, this study showed that the importance of CD2+ is reduced by high WBC counts. However, this research is limited by its retrospective design; therefore, further investigations on the relationships between CD2 and other molecular markers, bcr genotype, FLT3-ITD status, and treatment factors are warranted.

Flow cytometric immunophenotypic analysis can facilitate prompt diagnosis of APL. Although previous studies suggested the association of CD2+, CD34+, and CD56+ phenotypes with poor APL outcomes, the multivariate analysis has demonstrated that WBC count before treatment is the only known independent risk factor that predicts prognosis for this disease in this study population.

## Figures and Tables

**Table 1:** Immunophenotypic analysis of de novo APL patients [*n* (%)].

Antigens	Number (*N*)	Positive rate						Median of positive rate
		0–10%	10–20%	20–40%	40–60%	60–80%	80–100%	
cMPO	16	5 (31.3)		3 (18.8)	4 (25)	2 (12.5)	2 (12.5)	38.62
CD33	132	1 (0.8)		5 (3.8)	13 (9.8)	17 (12.9)	96 (72.7)	94.19
CD13	131	5 (2.9)		16 (9.2)	25 (14.4)	25 (14.4)	60 (34.5)	75.86
CD117	131	97 (74.0)		20 (15.3)	9 (6.9)	5 (3.8)	0 (0)	6.14
CD9	87	3 (3.4)		9 (10.3)	10 (11.5)	23 (26.4)	42 (48.3)	78.34
CD64	102	22 (21.6)		15 (14.7)	21 (20.6)	26 (25.5)	18 (17.6)	54.63
CD11b	99	89 (89.9)		8 (8.1)	0 (0)	1 (1.0)	1 (1.0)	1.98
HLA-DR	130	125 (96.2)		4 (3.1)	0	0	1 (0.8)	1.42
CD2	101	89 (88.1)		7 (6.9)	2 (2.0)	1 (1.0)	2 (2.0)	1.55
CD19	119	116 (97.5)		2 (1.7)	1 (0.8)	0	0	0.63
CD15	52	51 (98.1)		0	1 (1.9)	0	0	1.33
CD71	42	18 (42.9)		15 (35.7)	4 (9.5)	5 (11.9)	0 (0)	22.52
CD34	129	112 (86.8)	2 (1.6)	4 (3.1)	3 (2.3)	1 (0.8)	7 (5.4)	0.88
CD56	113	107 (94.7)	2 (1.7)	2 (1.7)	1 (0.9)	1 (0.9)	0	0.4

**Table 2:** Comparisons of clinical features and clinical outcomes between CD2+ APL and CD2− APL patients.

Group	CD2− APL	CD2+ APL	*P* value
Case number	89	12	
Age (years) (mean ± SD)	33.67 ± 12.88	30.50 ± 9.85	0.414
Gender (M/F)	49/40	8/4	0.446
WBC count before treatment (×10^9^/L) (mean ± SD)	15.06 ± 22.49	34.97 ± 57.6	0.028
CD13+ rate (%) (mean ± SD)	71.00 ± 25.23	71.07 ± 27.52	0.992
CD33+ rate (%) (mean ± SD)	83.96 ± 20.33	83.83 ± 19.24	0.984
CD117+ rate (%) (mean ± SD)	12.62 ± 17.42	12.05 ± 16.33	0.556
CD9+ rate (%) (mean ± SD)	73.60 ± 24.26	68.35 ± 28.47	0.705
CD64+ rate (%) (mean ± SD)	52.74 ± 26.96	49.21 ± 31.64	0.915
CD34+ rate (%) (mean ± SD)	3.63 ± 12.45	13.74 ± 16.42	0.006
CD56+ rate (%) (mean ± SD)	2.41 ± 6.89	0.53 ± 0.53	0.621
*Induction therapy *			
ATRA [*n* (%)]	18 (20.2)	4 (33.3)	
ATRA + chemotherapy [*n* (%)]	38 (42.7)	5 (41.7)	
ATRA + As_3_O_2 _[*n* (%)]	6 (6.7)	1 (8.3)	
ATRA + As_3_O_2_ + chemotherapy [*n* (%)]	27 (30.3)	2 (16.7)	
DS incidence [*n* (%)]	64 (28.0)	3 (25.0)	1.00
Early death [*n* (%)]	14 (15.7)	6 (50)	0.016
Hemorrhage [*n* (%)]	10 (71.4)	4 (66.7)	
Differentiation syndrome [*n* (%)]	2 (14.3)	1 (16.7)	
Infection [*n* (%)]	1 (7.1)	1 (16.7)	
Others [*n* (%)]	1 (7.1)	0 (0)	
CR rate [*n* (%)]	72 (91.1)	6 (50)	0.042
5-year OS [*n* (%)]	66 (74.2)	5 (41.7)	0.018
5-year relapse rate [*n* (%)]	5 (7.8)	1 (8.3)	1.00

CR: clinical response; DS: differentiation syndrome; OS: overall survival; WBC: white blood cell.

**Table 3:** Effect of CD2 on early death.

Dependent variables	Independent variables	*B*	SE	Wald	*P *value	OR	95% CI of OR
Early death	CD2+	1.466	0.741	3.918	0.048	4.333	1.015–18.508
	Constant	−1.689	0.314	28.914	0.000	0.185	

CI: confidence interval; SE: standard error; *B*: constant; OR: odds ratio; Wald: *χ* ^2^.

**Table 4:** Multivariate analysis with CD2, CD34, and CD56 expression and WBC count.

Dependent variables	Independent variables	*B*	SE	Wald	*P* value	OR	95% CI of OR
Early death	WBC count	0.019	0.009	4.439	0.035	1.1019	1.001–1.038
	Constant	−1.904	0.362	27.608	0.000		

Remission failure	WBC count	0.024	0.010	6.022	0.014	1.024	1.005–1.044
	Constant	−1.857	0.362	26.342	0.000		

5-year OS	WBC count	0.027	0.010	7.146	0.008	1.028	1.007–1.049
	Constant	−1.817	0.362	25.182	0.000	0.183	

CI: confidence interval; SE: standard error; WBC: white blood cell; OS: overall survival; *B*: constant; OR: odds ratio; Wald: *χ* ^2^.
